# 6-(4-Chloro­phen­yl)-2-(4-meth­oxy­phen­yl)-6,7-dihydro-4*H*-pyrazolo­[5,1-*c*][1,4]oxazine

**DOI:** 10.1107/S1600536811025906

**Published:** 2011-07-09

**Authors:** Liang-Wen Zheng, Bao-Xiang Zhao

**Affiliations:** a School of Chemistry and Chemical Engineering, Shandong University, Jinan 250100, People’s Republic of China

## Abstract

In the title compound, C_19_H_17_ClN_2_O_2_, the pyrazole ring is almost planar with a maximum deviation of 0.009 (3) Å and makes a dihedral angle of 8.96 (9)° with the oxazine ring. The dihedral angles between the pyrazole ring and the chlorine- and meth­oxy-substituted benzene rings are 50.95 (8) and 13.24 (9)°, respectively. An inter­molecular C—H⋯N hydrogen bond links the mol­ecules into infinite chains along the *a* axis. The crystal structure is further stabilized by C—H⋯π inter­actions.

## Related literature

For the pharmacological activity of pyrazole fused-heterocycles, see: Liu *et al.* (2011 ▶); Kumar *et al.* (2011 ▶); Guerrini *et al.* (2010 ▶). For related structures, see: Wei *et al.* (2007 ▶); Xie *et al.* (2009 ▶); Shimizu *et al.* (1990 ▶).
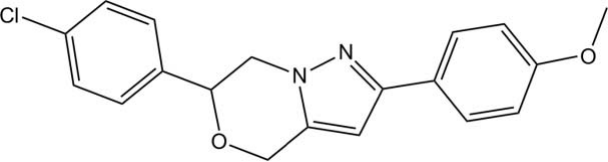


## Experimental

### 

#### Crystal data

C_19_H_17_ClN_2_O_2_
*M* *_r_* = 340.80
Monoclinic, 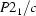

*a* = 6.0800 (8) Å
*b* = 34.224 (5) Å
*c* = 8.1217 (11) Å
β = 91.186 (3)°
*V* = 1689.6 (4) Å^3^
*Z* = 4
Mo *K*α radiation
μ = 0.24 mm^−1^
*T* = 298 K
0.15 × 0.12 × 0.10 mm

#### Data collection

Bruker SMART CCD area-detector diffractometer
Absorption correction: multi-scan (*SADABS*; Bruker, 2007 ▶) *T*_min_ = 0.965, *T*_max_ = 0.977
8891 measured reflections
2995 independent reflections
1620 reflections with *I* > 2σ(*I*)
*R*_int_ = 0.043

#### Refinement

*R*[*F* ^2^ > 2σ(*F* ^2^)] = 0.047
*wR*(*F* ^2^) = 0.123
*S* = 1.02
2995 reflections
218 parameters
1 restraint
H-atom parameters constrained
Δρ_max_ = 0.16 e Å^−3^
Δρ_min_ = −0.24 e Å^−3^

### 

Data collection: *APEX2* (Bruker, 2007 ▶); cell refinement: *APEX2* (Bruker, 2007 ▶); data reduction: *SAINT*; program(s) used to solve structure: *SHELXS97* (Sheldrick, 2008 ▶); program(s) used to refine structure: *SHELXL97* (Sheldrick, 2008 ▶); molecular graphics: *ORTEP-3* (Farrugia, 1997 ▶); software used to prepare material for publication: *SHELXTL* (Sheldrick, 2008 ▶).

## Supplementary Material

Crystal structure: contains datablock(s) I, global. DOI: 10.1107/S1600536811025906/ng5186sup1.cif

Structure factors: contains datablock(s) I. DOI: 10.1107/S1600536811025906/ng5186Isup2.hkl

Supplementary material file. DOI: 10.1107/S1600536811025906/ng5186Isup3.cml

Additional supplementary materials:  crystallographic information; 3D view; checkCIF report

## Figures and Tables

**Table 1:** Hydrogen-bond geometry (Å, °) *Cg*1 and *Cg*2 are the centroids of the N1,N2,C8–C10 and C14–C19 rings, respectively.

*D*—H⋯*A*	*D*—H	H⋯*A*	*D*⋯*A*	*D*—H⋯*A*
C11—H11*B*⋯N1^i^	0.97	2.47	3.419 (3)	167
C13—H13*B*⋯*Cg*1^ii^	0.97	2.86	3.762 (3)	156
C6—H6⋯*Cg*2^iii^	0.93	2.89	3.608 (3)	135

Symmetry codes: (i) 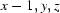; (ii) 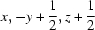; (iii) 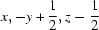.

**Fig. 1.**
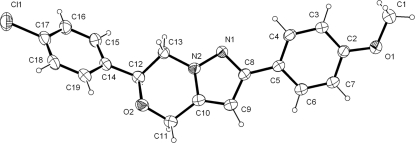
ORTEP view of compound (I), showing 25% probability displacement ellipsoids.

**Fig. 2.**
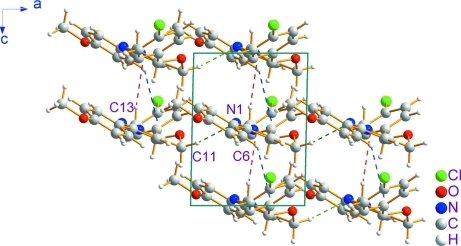
Packing diagram of compound (I). Hydrogen bonds are shown as dashed lines.

## supplementary crystallographic information

### Comment

A wide variety of heterocyclic systems have been explored for developing pharmaceutically important molecules. In the family of heterocyclic compounds, pyrazole derivatives possess important biological activity such as analgesic, anti-inflammatory, antipyretic, antiarrhythmic, tranquilizing, muscle relaxing, psychoanaleptic, anticonvulsant, monoamineoxidase inhibiting, antidiabetic and antibacterial activity. Pyrazole-fused heterocycles including pyrazolo[3,4-*b*]pyridine, pyrazolo[3,4-*d*]pyrimidine and pyrazolo[5,1-*b*][1,3]oxazine have attracted considerable attention. In the structure of raceme (I), two substituted benzene rings are bonded to pyrazolo[5,1-*c*][1,4]oxazine moiety at C8 and C12 as showed in Fig. 1. The torsion angle of C(1)–O(1)–C(2)–C(3) is -177.6 (2)° for compound (I), demonstrating that the methoxyl group is nearly planar in relation to the benzene ring. The moiety of oxazine ring is approximately planar with maximum mean plane deviation of -0.330 (2) Å for atom O2 and it is coplanar with the pyrazole ring, with a dihedral angle of only about 8.96 (9)°. The dihedral angles formed by chlorine substituted benzene ring and methoxy substituted benzene ring with pyrazole are 50.95 (8)° and 13.24 (9)°, respectively. Regarding the packing structure of compound (I), the C11–H11B···N1 hydrogen bond self-assembles the molecules into *C*(5) chains parallel to the *a* axis. Moreover, the structure is consolidated by C–H···π interactions between the aforementioned chains.

### Experimental

To a 100 ml round-bottomed flask equipped with a magnetic stirrer, 1-(4-chlorophenyl)-2-(5-(hydroxymethyl)-3-(4-methoxyphenyl)-1*H*-pyrazol-1-yl)ethanol (1.0 mmol) and 50% H_2_SO_4_ (3 drops) in 35 ml toluene were charged. The flask was stirred and heated at reflux for 4 h, until TLC indicated the end of the reaction. Solvent was removed and the resulting residue was partitioned with water and ethyl acetate. The organic layer was washed successively with brine and water and dried over MgSO_4_, then evaporated under reduced pressure to give a residue. Compound (I) was obtained without further purification in 84% yield. Crystals of (I) suitable for X-ray diffraction were obtained by slow evaporation of a solution of the solid in ethanol at room temperature for 3 days.

### Refinement

All the H atoms were positioned in their idealized geometries with C—H = 0.93–0.97Å and were refined using a riding model with *U*_iso_(H) = 1.5Ueq for methyl groups and with *U*_iso_(H) = 1.2Ueq for others.

### Crystal data

**Table:**

C_19_H_17_ClN_2_O_2_	*F*(000) = 712
*M**_r_* = 340.80	*D*_x_ = 1.340 Mg m^−^^3^*D*_m_ = 1.340 Mg m^−^^3^*D*_m_ measured by not measured
Monoclinic, *P*2_1_/*c*	Mo *K*α radiation, λ = 0.71073 Å
Hall symbol: -P 2ybc	Cell parameters from 1213 reflections
*a* = 6.0800 (8) Å	θ = 3.4–19.3°
*b* = 34.224 (5) Å	µ = 0.24 mm^−^^1^
*c* = 8.1217 (11) Å	*T* = 298 K
β = 91.186 (3)°	Block, colourless
*V* = 1689.6 (4) Å^3^	0.15 × 0.12 × 0.10 mm
*Z* = 4	

### Data collection

**Table:**

Bruker SMART CCD area-detector diffractometer	2995 independent reflections
Radiation source: fine-focus sealed tube	1620 reflections with *I* > 2σ(*I*)
graphite	*R*_int_ = 0.043
φ and ω scans	θ_max_ = 25.0°, θ_min_ = 2.4°
Absorption correction: multi-scan (*SADABS*; Bruker, 2007)	*h* = −7→7
*T*_min_ = 0.965, *T*_max_ = 0.977	*k* = −40→37
8891 measured reflections	*l* = −6→9

### Refinement

**Table:**

Refinement on *F*^2^	Primary atom site location: structure-invariant direct methods
Least-squares matrix: full	Secondary atom site location: difference Fourier map
*R*[*F*^2^ > 2σ(*F*^2^)] = 0.047	Hydrogen site location: inferred from neighbouring sites
*wR*(*F*^2^) = 0.123	H-atom parameters constrained
*S* = 1.02	*w* = 1/[σ^2^(*F*_o_^2^) + (0.0496*P*)^2^ + 0.0204*P*] where *P* = (*F*_o_^2^ + 2*F*_c_^2^)/3
2995 reflections	(Δ/σ)_max_ < 0.001
218 parameters	Δρ_max_ = 0.16 e Å^−^^3^
1 restraint	Δρ_min_ = −0.24 e Å^−^^3^

### Special details

**Table:**

Geometry. All e.s.d.'s (except the e.s.d. in the dihedral angle between two l.s. planes) are estimated using the full covariance matrix. The cell e.s.d.'s are taken into account individually in the estimation of e.s.d.'s in distances, angles and torsion angles; correlations between e.s.d.'s in cell parameters are only used when they are defined by crystal symmetry. An approximate (isotropic) treatment of cell e.s.d.'s is used for estimating e.s.d.'s involving l.s. planes.
Refinement. Refinement of *F*^2^ against ALL reflections. The weighted *R*-factor *wR* and goodness of fit *S* are based on *F*^2^, conventional *R*-factors *R* are based on *F*, with *F* set to zero for negative *F*^2^. The threshold expression of *F*^2^ > σ(*F*^2^) is used only for calculating *R*-factors(gt) *etc*. and is not relevant to the choice of reflections for refinement. *R*-factors based on *F*^2^ are statistically about twice as large as those based on *F*, and *R*- factors based on ALL data will be even larger.

### Fractional atomic coordinates and isotropic or equivalent isotropic displacement parameters (Å^2^)

**Table:**

	*x*	*y*	*z*	*U*_iso_*/*U*_eq_	
Cl1	0.30584 (16)	0.48228 (2)	0.68319 (12)	0.1196 (4)	
O1	0.9536 (3)	0.04587 (5)	0.5717 (3)	0.0913 (6)	
O2	0.1081 (3)	0.29880 (5)	0.4635 (2)	0.0731 (5)	
N1	0.6176 (3)	0.22476 (6)	0.5415 (3)	0.0634 (6)	
N2	0.4637 (3)	0.25198 (6)	0.4972 (2)	0.0616 (6)	
C1	1.1487 (5)	0.04055 (9)	0.6669 (4)	0.1062 (11)	
H1A	1.2629	0.0568	0.6243	0.159*	
H1B	1.1932	0.0137	0.6617	0.159*	
H1C	1.1224	0.0475	0.7792	0.159*	
C2	0.8607 (4)	0.08212 (8)	0.5635 (3)	0.0664 (7)	
C3	0.9452 (4)	0.11536 (8)	0.6365 (3)	0.0694 (8)	
H3	1.0751	0.1142	0.6988	0.083*	
C4	0.8355 (4)	0.15036 (8)	0.6166 (3)	0.0683 (7)	
H4	0.8946	0.1727	0.6652	0.082*	
C5	0.6404 (4)	0.15335 (7)	0.5267 (3)	0.0588 (7)	
C6	0.5596 (5)	0.11944 (8)	0.4548 (4)	0.0826 (9)	
H6	0.4298	0.1204	0.3924	0.099*	
C7	0.6668 (5)	0.08457 (8)	0.4738 (4)	0.0857 (9)	
H7	0.6078	0.0622	0.4254	0.103*	
C8	0.5221 (4)	0.19052 (7)	0.5029 (3)	0.0576 (6)	
C9	0.3076 (4)	0.19647 (8)	0.4394 (3)	0.0644 (7)	
H9	0.2073	0.1774	0.4060	0.077*	
C10	0.2768 (4)	0.23584 (8)	0.4367 (3)	0.0610 (7)	
C11	0.0911 (4)	0.26170 (8)	0.3834 (4)	0.0729 (8)	
H11A	0.0938	0.2653	0.2650	0.087*	
H11B	−0.0476	0.2495	0.4104	0.087*	
C12	0.3161 (4)	0.31656 (7)	0.4375 (3)	0.0659 (7)	
H12	0.3449	0.3167	0.3192	0.079*	
C13	0.4982 (4)	0.29340 (7)	0.5270 (3)	0.0661 (7)	
H13A	0.6408	0.3012	0.4868	0.079*	
H13B	0.4951	0.2987	0.6442	0.079*	
C14	0.3092 (4)	0.35817 (8)	0.4984 (3)	0.0626 (7)	
C15	0.4814 (5)	0.38322 (8)	0.4675 (3)	0.0739 (8)	
H15	0.6001	0.3741	0.4082	0.089*	
C16	0.4825 (5)	0.42145 (8)	0.5224 (4)	0.0818 (9)	
H16	0.6003	0.4378	0.5005	0.098*	
C17	0.3068 (5)	0.43484 (8)	0.6096 (4)	0.0790 (8)	
C18	0.1343 (5)	0.41086 (9)	0.6415 (3)	0.0797 (8)	
H18	0.0157	0.4203	0.7001	0.096*	
C19	0.1340 (4)	0.37241 (8)	0.5870 (3)	0.0720 (8)	
H19	0.0160	0.3562	0.6100	0.086*	

### Atomic displacement parameters (Å^2^)

**Table:**

	*U*^11^	*U*^22^	*U*^33^	*U*^12^	*U*^13^	*U*^23^
Cl1	0.1541 (9)	0.0772 (6)	0.1269 (8)	0.0344 (6)	−0.0109 (7)	−0.0143 (5)
O1	0.0986 (15)	0.0626 (13)	0.1113 (17)	0.0022 (11)	−0.0298 (13)	−0.0065 (11)
O2	0.0562 (11)	0.0821 (13)	0.0810 (14)	0.0034 (9)	0.0026 (9)	−0.0061 (10)
N1	0.0566 (12)	0.0586 (14)	0.0750 (15)	0.0020 (11)	0.0004 (11)	−0.0024 (11)
N2	0.0523 (12)	0.0614 (14)	0.0712 (15)	−0.0045 (11)	0.0034 (11)	−0.0028 (11)
C1	0.091 (2)	0.082 (2)	0.144 (3)	0.0093 (18)	−0.020 (2)	0.003 (2)
C2	0.0752 (19)	0.0569 (18)	0.0669 (19)	−0.0041 (14)	−0.0046 (15)	0.0004 (14)
C3	0.0641 (17)	0.0671 (19)	0.077 (2)	−0.0056 (14)	−0.0116 (14)	−0.0058 (15)
C4	0.0655 (17)	0.0610 (18)	0.078 (2)	−0.0139 (14)	−0.0026 (15)	−0.0078 (14)
C5	0.0616 (16)	0.0598 (17)	0.0548 (17)	−0.0096 (13)	0.0021 (13)	−0.0013 (13)
C6	0.083 (2)	0.070 (2)	0.094 (2)	−0.0036 (17)	−0.0283 (17)	−0.0106 (17)
C7	0.091 (2)	0.065 (2)	0.099 (2)	−0.0084 (16)	−0.0333 (19)	−0.0116 (16)
C8	0.0619 (17)	0.0588 (17)	0.0522 (16)	−0.0094 (13)	0.0047 (13)	−0.0021 (12)
C9	0.0611 (17)	0.074 (2)	0.0584 (18)	−0.0141 (14)	0.0027 (14)	−0.0028 (13)
C10	0.0566 (16)	0.0723 (19)	0.0544 (17)	−0.0065 (14)	0.0063 (13)	−0.0046 (13)
C11	0.0615 (16)	0.081 (2)	0.076 (2)	−0.0010 (15)	−0.0031 (14)	−0.0012 (16)
C12	0.0608 (16)	0.0746 (19)	0.0626 (18)	0.0013 (14)	0.0091 (14)	0.0027 (14)
C13	0.0558 (16)	0.0622 (17)	0.081 (2)	0.0028 (13)	0.0080 (14)	−0.0054 (14)
C14	0.0635 (16)	0.0686 (18)	0.0558 (17)	0.0132 (15)	0.0004 (13)	0.0026 (13)
C15	0.0754 (19)	0.072 (2)	0.075 (2)	0.0103 (16)	0.0104 (15)	0.0025 (15)
C16	0.085 (2)	0.067 (2)	0.094 (2)	0.0066 (16)	0.0010 (18)	0.0078 (16)
C17	0.091 (2)	0.074 (2)	0.071 (2)	0.0246 (14)	−0.0134 (17)	0.0015 (15)
C18	0.0781 (19)	0.085 (2)	0.075 (2)	0.0335 (13)	0.0018 (16)	−0.0026 (16)
C19	0.0664 (17)	0.083 (2)	0.0668 (19)	0.0155 (15)	0.0013 (15)	0.0057 (15)

### Geometric parameters (Å, °)

**Table:**

Cl1—C17	1.730 (3)	C7—H7	0.9300
O1—C2	1.364 (3)	C8—C9	1.408 (3)
O1—C1	1.414 (3)	C9—C10	1.361 (3)
O2—C12	1.423 (3)	C9—H9	0.9300
O2—C11	1.429 (3)	C10—C11	1.491 (3)
N1—C8	1.342 (3)	C11—H11A	0.9700
N1—N2	1.363 (2)	C11—H11B	0.9700
N2—C10	1.347 (3)	C12—C14	1.508 (3)
N2—C13	1.452 (3)	C12—C13	1.533 (3)
C1—H1A	0.9600	C12—H12	0.9800
C1—H1B	0.9600	C13—H13A	0.9700
C1—H1C	0.9600	C13—H13B	0.9700
C2—C7	1.375 (3)	C14—C15	1.380 (3)
C2—C3	1.378 (3)	C14—C19	1.386 (3)
C3—C4	1.379 (3)	C15—C16	1.382 (3)
C3—H3	0.9300	C15—H15	0.9300
C4—C5	1.384 (3)	C16—C17	1.373 (4)
C4—H4	0.9300	C16—H16	0.9300
C5—C6	1.384 (3)	C17—C18	1.361 (4)
C5—C8	1.472 (3)	C18—C19	1.388 (4)
C6—C7	1.367 (3)	C18—H18	0.9300
C6—H6	0.9300	C19—H19	0.9300
			
C2—O1—C1	119.1 (2)	C9—C10—C11	134.1 (2)
C12—O2—C11	111.61 (19)	O2—C11—C10	110.4 (2)
C8—N1—N2	104.12 (19)	O2—C11—H11A	109.6
C10—N2—N1	112.7 (2)	C10—C11—H11A	109.6
C10—N2—C13	125.4 (2)	O2—C11—H11B	109.6
N1—N2—C13	121.79 (19)	C10—C11—H11B	109.6
O1—C1—H1A	109.5	H11A—C11—H11B	108.1
O1—C1—H1B	109.5	O2—C12—C14	108.9 (2)
H1A—C1—H1B	109.5	O2—C12—C13	110.1 (2)
O1—C1—H1C	109.5	C14—C12—C13	111.0 (2)
H1A—C1—H1C	109.5	O2—C12—H12	108.9
H1B—C1—H1C	109.5	C14—C12—H12	108.9
O1—C2—C7	115.5 (2)	C13—C12—H12	108.9
O1—C2—C3	125.4 (3)	N2—C13—C12	109.0 (2)
C7—C2—C3	119.1 (3)	N2—C13—H13A	109.9
C2—C3—C4	119.4 (2)	C12—C13—H13A	109.9
C2—C3—H3	120.3	N2—C13—H13B	109.9
C4—C3—H3	120.3	C12—C13—H13B	109.9
C3—C4—C5	122.2 (2)	H13A—C13—H13B	108.3
C3—C4—H4	118.9	C15—C14—C19	118.0 (3)
C5—C4—H4	118.9	C15—C14—C12	120.1 (2)
C4—C5—C6	117.0 (2)	C19—C14—C12	121.9 (3)
C4—C5—C8	123.0 (2)	C14—C15—C16	121.9 (3)
C6—C5—C8	120.1 (2)	C14—C15—H15	119.1
C7—C6—C5	121.3 (3)	C16—C15—H15	119.1
C7—C6—H6	119.3	C17—C16—C15	118.9 (3)
C5—C6—H6	119.3	C17—C16—H16	120.5
C6—C7—C2	121.0 (3)	C15—C16—H16	120.5
C6—C7—H7	119.5	C18—C17—C16	120.5 (3)
C2—C7—H7	119.5	C18—C17—Cl1	119.5 (2)
N1—C8—C9	110.6 (2)	C16—C17—Cl1	120.0 (3)
N1—C8—C5	121.1 (2)	C17—C18—C19	120.5 (3)
C9—C8—C5	128.3 (2)	C17—C18—H18	119.8
C10—C9—C8	106.0 (2)	C19—C18—H18	119.8
C10—C9—H9	127.0	C14—C19—C18	120.2 (3)
C8—C9—H9	127.0	C14—C19—H19	119.9
N2—C10—C9	106.6 (2)	C18—C19—H19	119.9
N2—C10—C11	119.4 (2)		
			
C8—N1—N2—C10	1.4 (3)	C8—C9—C10—N2	−0.5 (3)
C8—N1—N2—C13	176.7 (2)	C8—C9—C10—C11	179.3 (3)
C1—O1—C2—C7	−177.6 (3)	C12—O2—C11—C10	54.9 (3)
C1—O1—C2—C3	2.4 (4)	N2—C10—C11—O2	−22.8 (3)
O1—C2—C3—C4	179.2 (2)	C9—C10—C11—O2	157.4 (3)
C7—C2—C3—C4	−0.8 (4)	C11—O2—C12—C14	170.0 (2)
C2—C3—C4—C5	0.8 (4)	C11—O2—C12—C13	−68.1 (3)
C3—C4—C5—C6	−0.7 (4)	C10—N2—C13—C12	−15.0 (3)
C3—C4—C5—C8	−179.5 (2)	N1—N2—C13—C12	170.3 (2)
C4—C5—C6—C7	0.8 (4)	O2—C12—C13—N2	44.8 (3)
C8—C5—C6—C7	179.5 (3)	C14—C12—C13—N2	165.4 (2)
C5—C6—C7—C2	−0.8 (5)	O2—C12—C14—C15	−171.6 (2)
O1—C2—C7—C6	−179.1 (3)	C13—C12—C14—C15	67.1 (3)
C3—C2—C7—C6	0.8 (5)	O2—C12—C14—C19	9.0 (3)
N2—N1—C8—C9	−1.7 (3)	C13—C12—C14—C19	−112.4 (3)
N2—N1—C8—C5	177.7 (2)	C19—C14—C15—C16	0.0 (4)
C4—C5—C8—N1	12.5 (4)	C12—C14—C15—C16	−179.4 (2)
C6—C5—C8—N1	−166.2 (2)	C14—C15—C16—C17	−0.2 (4)
C4—C5—C8—C9	−168.2 (2)	C15—C16—C17—C18	0.0 (4)
C6—C5—C8—C9	13.1 (4)	C15—C16—C17—Cl1	178.8 (2)
N1—C8—C9—C10	1.4 (3)	C16—C17—C18—C19	0.4 (4)
C5—C8—C9—C10	−177.9 (2)	Cl1—C17—C18—C19	−178.45 (19)
N1—N2—C10—C9	−0.6 (3)	C15—C14—C19—C18	0.3 (4)
C13—N2—C10—C9	−175.6 (2)	C12—C14—C19—C18	179.7 (2)
N1—N2—C10—C11	179.6 (2)	C17—C18—C19—C14	−0.5 (4)
C13—N2—C10—C11	4.5 (4)		

### Hydrogen-bond geometry (Å, °)

**Table:**

Cg1 and Cg2 are the centroids of the N1,N2,C8–C10 and C14–C19 rings, respectively.

**Table:**

*D*—H···*A*	*D*—H	H···*A*	*D*···*A*	*D*—H···*A*
C11—H11B···N1^i^	0.97	2.47	3.419 (3)	167
C13—H13B···Cg1^ii^	0.97	2.86	3.762 (3)	156
C6—H6···Cg2^iii^	0.93	2.89	3.608 (3)	135

Symmetry codes: (i) *x*−1, *y*, *z*; (ii) *x*, −*y*+1/2, *z*+1/2; (iii) *x*, −*y*+1/2, *z*−1/2.
